# The examination of detrusor underactivity in multiple system atrophy

**DOI:** 10.3389/fneur.2024.1460379

**Published:** 2024-09-17

**Authors:** Tatsuya Yamamoto, Ryuji Sakakibara, Tomoyuki Uchiyama, Satoshi Kuwabara

**Affiliations:** ^1^Department of Neurology, Chiba University Graduate School of Medicine, Chiba, Japan; ^2^Department of Rehabilitation Sciences, Chiba Prefectural University of Health Sciences, Chiba, Japan; ^3^Neurology Clinic Tsudanuma, Chiba, Japan; ^4^Department of Neurology, International University of Health and Welfare, Chiba, Japan

**Keywords:** multiple system atrophy, urodynamic study, bladder contractility index, post void residuals, voided percentage

## Abstract

**Aims:**

The urinary dysfunction in multiple system atrophy (MSA) is characterized by large post-void residuals (PVR) due to impaired bladder contractility. However, the evaluations of bladder contractility are not well validated in MSA. Because the bladder contractility index can be generally represented as Pdet Qmax (detrusor pressure at maximum urinary flow rate) + kQmax (maximum urinary flow rate), we aim to examine which “k” value is suitable for representing bladder contractility concerning its correlations to PVR and voided percentage (VOID%).

**Methods:**

We retrospectively reviewed 133 patients with MSA (74 males, 59 females, mean disease duration 3.2 years) who underwent an urodynamic study. We calculated bladder contractility using the formula PIP_k_ = Pdet Qmax + kQmax by increasing the “k” value from 0.1 to 10 by increments of 0.1. We calculated the correlations between each PIP_k_ (k = 0.1–10.0) and PVR and VOID%.

**Results:**

The correlational coefficients between PIP_k_ and VOID% were larger than those between PIP_k_ and PVR. The correlational coefficients between PIP_k_ and VOID% reached a plateau level at a “k” value >5.0 in male patients, suggesting that currently used formulas such as Pdet Qmax + 5Qmax for males might be appropriate for male MSA patients. However, the correlational coefficients between PIP_k_ and VOID% reached a plateau level in female patients when the “k” values were >6.0, which might overestimate bladder contractility in female patients.

**Conclusion:**

Although currently used formulas such as Pdet Qmax + 5Qmax might be appropriate for male MSA patients, formulas for female patients need further evaluation.

## Introduction

Multiple system atrophy (MSA) is clinically characterized by the concurrent presence of autonomic, extrapyramidal, and cerebellar dysfunctions (1). Among its prevalent and severe symptoms, lower urinary tract dysfunction stands out (2, 3). Voiding issues are particularly widespread, with a majority of MSA patients exhibiting significant post-void residual (PVR) volume (4, 5). This elevated PVR in MSA cases stems from compromised detrusor contractility, attributed to the degeneration of the parasympathetic nucleus within the sacral cord that innervates the bladder (6). In clinical practice, elevated PVR suggests the presence of detrusor underactivity or bladder outlet obstruction. Urodynamic study (UDS) is useful in differentiating detrusor underactivity from bladder outlet obstruction. Detrusor underactivity is characterized by reduced strength and/or duration of detrusor contraction, resulting in prolonged bladder emptying and/or a failure to achieve complete bladder emptying within a normal period during UDS (7). Nonetheless, the precise criteria for definitively diagnosing detrusor underactivity through urodynamic findings in individuals with MSA remain inadequately established. However, elevated PVR caused by detrusor underactivity is a significant clinical hallmark in the Movement disorder society (MDS) criteria for the diagnosis of MSA (8), the established criteria for diagnosing detrusor underactivity in MSA are essential.

A recent review highlighted that the term “contractility” currently lacks a formal definition in the context of bladder voiding function (9). The detrusor contractility may relate to detrusor myocyte, autonomic nervous system innervating bladder, and cognitive functions (9). However, the right method to measure contractility is still problematic and there are no definitive criteria to evaluate detrusor contractility considering the previous factors related to it.

In clinical practice, detrusor underactivity is defined as a failure to create sufficient pressure and/or sufficient sustained pressure to empty the bladder normally and completely (7, 9). On the other hand, the expulsive dynamics of the detrusor are usually evaluated by detrusor pressure and flow rate obtained from the UDS.

Multiple approaches exist for assessing detrusor contraction. While Schäfer’s nomogram finds popularity among men with benign prostatic enlargement, its applicability to women is limited (10). In contrast, the Watts factor is applicable to both males and females. However, the need for complex calculations limits its use in clinical practice (11). In general, the bladder contractility index (BCI), which relies on the projected isovolumetric pressure (PIP), is denoted as PIP_k_ = Pdet Qmax + kQmax in its description. Schäfer provided the equation to the following form PIP_5_ = Pdet Qmax + 5Qmax, where he used a fixed K value equal to 5 for male patients. On the other hand, some studies recommend using PIP_1_ = Pdet Qmax + Qmax for older women instead of using PIP_5_ as it might overestimate bladder contractility (9).

However, currently used formulas such as PdetQmax + 5Qmax for male patients and Pdet Qmax + Qmax for female patients is not validated for patients with neurological dysfunctions including MSA. Although elucidating detrusor contractility which significantly affects PVR is crucial in the clinical practice of MSA, we do not know whether the currently used formula is suitable for evaluating detrusor contractility in MSA patients. Therefore, the purpose of this study is to elucidate the appropriate formula representing detrusor contractility with respect to its correlation with PVR or voiding efficiency in MSA patients.

We do not know which k value is appropriate for both genders to evaluate bladder contractility in patients with MSA. It is also plausible that the k value might change depending on the cause of detrusor underactivity or gender. Therefore, the accurate evaluation of bladder contractility is important for both diagnosis and understanding of pathophysiological mechanisms of large PVR in patients with MSA.

Our objective is to determine the most appropriate “k” value for representing bladder contractility in relation to its associations with PVR and voided percentage (VOID%) during the pressure-flow study in patients with MSA.

## Methods

The patients were included in the study if they were diagnosed as possible or probable MSA according to Gilman’s second consensus criteria (12). The patients were referred for a UDS because all MSA patients in this study had some lower urinary tract symptoms, such as urinary frequency, urinary urgency, sensation of incomplete emptying, intermittency, weak stream, and voiding difficulty. UDS was performed to clarify the causes of both storage and voiding symptoms.

Patients were omitted if they were unable to urinate during the pressure-flow study, had concurrent urological conditions like benign prostatic hyperplasia, stress urinary incontinence or met the criteria for “obstruction” as per the ICS nomogram in male participants. The patients who had recurrent urinary tract infections and received clean intermittent catheterization were excluded in this study.

### UDS

A skilled neurologist and urologist, well-versed in the principles and outcomes of free flowmetry and Urodynamic Studies (UDS), performed the UDS procedure. The neurologist evaluated urinary dysfunctions and their associated neurological dysfunction. Firstly, we conducted free flowmetry to calculate the maximum and average flow rates even before the pressure-flow study. Then, transurethral catheterization was implemented to measure the PVR after calculating the voided volume. Additionally, we employed an urodynamic system (Janus; Life-Tec Inc., Houston, TX, USA) alongside an electromyography (EMG) device (Neuropack Sigma; Nihon Kohden Inc., Tokyo, Japan) to perform electromyographic cystometry. During the measurements, we place a coaxial needle electrode into the external anal sphincter (EAS) muscles to capture anal sphincter EMG activity. For water (saline) cystometry, an 8F double-lumen catheter is introduced through the urethra while the patient is in a seated position. The infusion rate during the procedure was 50 mL/min. Simultaneously, a balloon catheter was utilized to measure rectal pressure. Subsequently, this pressure was electronically deducted from the intravesical pressure. The next study after water cystometry was the pressure-flow study. The sitting position and the same environment were used in both procedures; free flowmetry and the pressure-flow study. The neurologists and urologists reviewed each flow study. However, we removed any erroneously elevated Qmax values resulting from abdominal straining.

Unusual urodynamic results in the storage phase encompassed detrusor overactivity, characterized by involuntary contractions of the detrusor muscle during storage. Within this storage phase, the quantifiable measures encompassed bladder volume at the first desire to void (FDV). An FDV below 100 mL was deemed abnormal. The volume at which the patient can no longer delay micturition is called maximum cystometric capacity. During the voiding phase, impaired bladder contractility was considered as an abnormal urodynamic finding. The Schäfer nomogram evaluated the degree of detrusor contraction and categorized them as “weak” and “very weak” contractility for men. Detrusor sphincter dyssynergia (DSD) was defined as a detrusor contraction concurrent with an involuntary contraction of the periurethral striated muscle as evaluated by EAS-EMG. All employed methodologies, definitions, and units are in accordance with the guidelines outlined by the International Continence Society (13).

### EAS EMG

Under audio guidance, we introduced a single-use concentric needle electrode (needle diameter: 0.46 mm; Alpine Biomed, Skovlunde, Denmark) into the outermost layer of the anal sphincter. Two needles were inserted into the right and left sphincter muscles at positions (5 o’clock) and (7 o’clock), respectively. Subsequently, we conducted an analysis of motor unit potentials (MUPs), examining five MUPs on each side. The placement of the needle electrode was adjusted until consistent firing patterns of 3–5 MUPs. The ascent duration of the MUP ranged from 300 to 500 μs. We recorded MUP with a gain of 100 μV at 5 ms/div at 1 cm from the anal orifice and with 3–6 mm depth. The amplifier filter was set at 5–10 kHz. The procedure for conducting External Anal Sphincter EMG (EAS-EMG) and the standards for diagnosing neurogenic alterations in EAS-EMG were akin to those applied in a prior investigation (4).

VOID% is characterized by the proportion of the volume voided to the combined bladder volume, which includes both the voided volume and the PVR volume.

We computed diverse PIP values using the formula PIP_k_ = Pdet Qmax + kQmax by incrementing the “k” value from 0.1 to 10 in steps of 0.1. Subsequently, we determined the correlation coefficients between each PIP_k_ (k = 0.1–10.0) and PVR. Additionally, we calculated the correlation coefficients between each PIP_k_ (k = 0.1–10.0) and VOID%. We then constructed a graph displaying the correlation coefficients between PIP_k_ and PVR against the “k” value, aiming to identify the “k” value associated with the strongest correlation between PIP_k_ and PVR. We also plotted the correlational coefficients between PIP_k_ and VOID% against the “k” value to determine which “k” value has the largest correlational coefficients between PIP_k_ and VOID%. We calculated the bladder outlet obstruction index (BOOI) using the formula BOOI = Pdet Qmax − 2Qmax in male patients.

### Statistical analysis

We expressed data using mean ± standard error. SPSS Version 28.0 (IBM, Armonk, USA) was used for all statistical analyses in this study. The student’s t-test was the test of choice to compare urodynamic parameters between males and females. We employed Spearman’s correlation coefficient to examine the connection between PVR/VOID% and PIP_k_ in both males and females. The sample size was calculated with an expected Spearman’s correlation coefficient of 0.5 or more and a test power of 0.8 and was 33 patients. The significant *p*-value was defined as 0.05.

## Results

We retrospectively reviewed 109 patients with MSA (50 males: 64.4 ± 0.6 yrs., 59 females: 62.2 ± 0.8 yrs., mean disease duration 3.2 years) who underwent UDS. Data was collected from September 1, 2018, to December 31, 2023, in Chiba University Hospital, Chiba, Japan. Seventy patients were classified as MSA-cerebellar type (MSA-C), whereas the remaining 39 patients were classified as MSA-parkinsonian type (MSA-P). Nineteen patients used alpha-blockers to relieve voiding dysfunction. Ten patients used anticholinergics, and one patient used a beta-3 adrenoceptor agonist to relieve storage symptoms. PVR was significantly higher in male patients (231 ± 21 mL) than that in females (162 ± 18 mL) (*p* < 0.01). VOID% in male patients (41.4% ± 4.1%) was, on the other hand, significantly smaller than in females (51.0% ± 4.2%) (*p* < 0.01). In males, the average Pdet Qmax was 38.9 ± 2.0 cmH_2_O, while the mean Qmax during the pressure-flow study stood at 7.7 ± 1.0 mL/s. As for female patients, the mean Pdet Qmax amounted to 26.0 ± 1.7 cmH_2_O, and the mean Qmax during the pressure-flow study was 9.2 ± 1.0 mL/s. As a result, the mean Pdet Qmax was significantly larger and Q max was significantly smaller in male patients than in female patients (*p* < 0.01). FDV, maximum cystometric capacity, prevalence of detrusor overactivity, and detrusor sphincter dyssynergia are represented in Table 1.

**Table 1:** Urodynamic parameters in male and female MSA patients.

	Male (*n* = 74)	Female (*n* = 59)	*p* value
PVR (mL)	231 ± 21	162 ± 18	<0.01
Void%	41.4 ± 4.1	51.0 ± 4.2	<0.01
Pdet Qmax (cmH_2_O)	38.9 ± 2.0	26.0 ± 1.7	<0.01
Qmax (mL/s)	7.7 ± 1.0	9.2 ± 1.0	<0.01

PVR, post-void residual volume; Void%, voided percentage.

**Figure 1:**
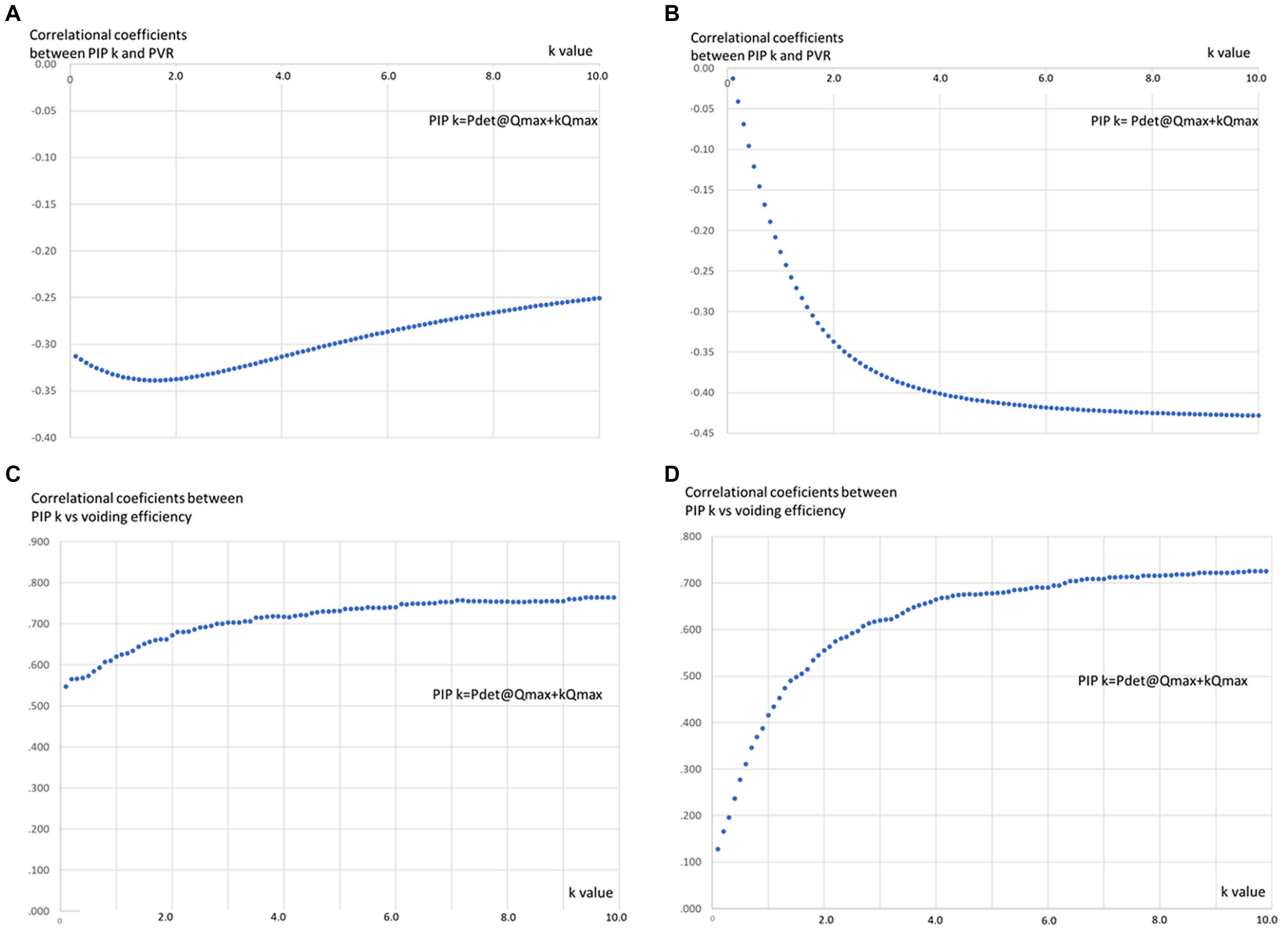
The relationships between “k” value and correlational coefficients between PIPk and PVR and VOID% in MSA patients. **(A)** In male patients, there was a significant negative correlation (*p* < 0.05) between PIP k and PVR during the pressure-flow study in all “k” values >0.1 and showed largest negative correlational coefficients when “k” value is 2.0. **(B)** A significant negative correlation (*p* < 0.05) between PIPk and PVR during the pressure-flow study was observed when the “k” values were >1.2 in female patients and correlational coefficients reached plateau level when “k” value is a value >6.0 in female patients. **(C)** PIPk and VOID% demonstrated significant positive correlations (*p* < 0.05) in male patients with all “k” values and reached plateau level when the “k” value became >5.0. **(D)** There were significant positive correlations between PIP k and VOID% in female patients when the “k” value was >0.5 which reached a plateau level at a “k” value >6.0.

The absolute value of correlational coefficients in each PIP_k_ (k = 0.1–10.0) and VOID% was larger than those between each PIP_k_ (k = 0.1–10.0) and PVR, suggesting that VOID% might be more appropriate than PVR in evaluating the ability of PIP_k_. Among male patients, a notable inverse correlation (*p* < 0.05) was observed between PIP_k_ and PVR throughout the pressure-flow study, holding true for all “k” values greater than 0.1. The most substantial negative correlation coefficients were evident when the “k” value was 2.0 (Figure 1A). Furthermore, in female patients, a noteworthy adverse correlation (*p* < 0.05) between PIP_k_ and PVR was noted throughout the pressure-flow study, particularly when “k” values exceeded 1.2. The correlation coefficients reached a plateau level with “k” values greater than 6.0 (Figure 1B).

On the other hand, PIP_k_ and VOID% demonstrated significant positive correlations (*p* < 0.05) in male patients with all “k” values and reached a plateau level when the “k” value became >5.0 (Figure 1C). However, significant positive correlations between PIP_k_ and VOID% were evident among female patients when the “k” value was >0.5 which reached a plateau level at a “k” value >6 (Figure 1D). The absolute value of correlational coefficients between PIP_k_ and VOID% was significantly larger than that between PIP_k_ and PVR in male and female patients.

No correlations were observed between BOOI and PVR (*r* = −0.243, *p* = 0.096) and VOID% (*r* = 0.182, *p* = 0.215) in male patients.

## Discussion

The present study examined the appropriate “k” value of PIP for representing bladder contractility and its correlation to PVR/VOID% in MSA males and females. Our results suggested that the absolute value of correlational coefficients between PIP_k_ and VOID% was significantly larger than that between PIP_k_ and PVR in patients with MSA in both genders. That may suggest the validity of VOID% as a more appropriate measure than PVR in terms of its relation to bladder contractility in patients with MSA. The present result suggested that a “k” value = 5 is suitable for MSA male patients, suggesting that the currently used formula “PIP_5_ = Pdet Qmax + 5Qmax “might be applicable to MSA male patients. Although “k” value = 6 might be suitable for its correlation to PIP_k_ in female patients, “k” value = 6 might also result in overestimating bladder contractility in females, suggesting that appropriate k value for MSA female patients could not be elucidated in this study.

In clinical practice, the degree of voiding dysfunction can be assessed by PVR/VOID%. However, high PVR/low VOID% may be caused by detrusor underactivity or BOO and should be differentiated by UDS findings. Because detrusor undeactivity is prevalent in many neurological diseases, including MSA, reliable methods to estimate detrusor contractility are needed. Detrusor contractility is regulated by the sacral parasympathetic nucleus which is usually degenerated in MSA. Therefore, accurate evaluation of detrusor contractility led to the evaluation of the sacral parasympathetic nucleus in MSA. However, currently available formulas such as BCI (PIP5) and PIP1 to estimate detrusor contractility have not been validated in MSA. Therefore, we investigated which k value of PIP k is suitable for estimating detrusor contractility in terms of its correlation with PVR/VOID%.

The associations between the “k” value and the corresponding correlation coefficients of PIP_k_ with PVR/VOID% displayed distinct variations between male and female patients with MSA. Concerning the correlation between PIP_k_ and VOID%, the correlational coefficient was relatively larger (*r* = 0.547, *p* < 0.01) in male patients compared to female patients (*r* = 0.128, *p* = 0.331) when the “k” value was 0.1. Correlational coefficients between PIP_k_ and VOID% gradually increased in male patients as the “k” value increased to reach a plateau level at a “k” value >5.0. On the contrary, the correlational coefficient between PIP_k_ and VOID% behaves in female patients the same way as in males but it reaches the plateau at a “k” value >6.0. Although the correlational coefficient between PIP_k_ and PVR was negative, the relationship between PIP_k_ and PVR was basically like the relationships between PIP_k_ and VOID%.

Considering that the correlation coefficients between PIP_k_ and PVR/VOID% reached a plateau beyond a “k” value of 5.0, utilizing PIP_5_ (where PIP_5_ = Pdet Qmax + 5Qmax) could be suitable for assessing detrusor contractility in male patients with MSA. Additionally, as PIP_5_ is widely used as BCI in evaluating detrusor contractility in male patients with or without neurological disorders (14, 15), it might be acceptable that detrusor contractility in male patients with MSA could be examined by using Pdet Qmax + 5Qmax.

On the contrary, the correlational coefficients between PIP_k_ and PVR/VOID% reached the plateau level at a “k” value exceeding 6.0 in patients with MSA from the female group. Therefore, the exceptionally large “k” value might be appropriate in terms of its relation to PVR/VOID% in this group. Although the examinations of detrusor underactivity in female patients with or without neurological disorders are controversial, for older women, PIP1 = Pdet Qmax + Qmax was employed instead of PIP_5_, as the latter might lead to an overestimation of detrusor contractility in females (7). It is postulated that “k” values >6.0 in this study should be further explored in the future. As a result, it is difficult to conclude the appropriate “k” value in female patients with MSA in this study. One possible reason for the difference between male and female patients is that the urethral function contributes to voiding function, especially in female patients. It is well known that in some women, the marked decrease in urethral resistance during urination determines urination with minimal variation in detrusor pressure, despite the occurrence of detrusor contraction. Another formula representing detrusor contractility in female MSA patients needs to be invented. However, the definition and diagnosing detrusor underactivity have not been validated even in the field of urology, and establishing a formula representing detrusor contractility in female MSA patients needs further investigation (16).

The influence of intra-abdominal pressure should also be mentioned in this study. It is not uncommon for patients to be able to void using sustained intra-abdominal pressure rather than detrusor pressure. When we examined Pdet max and Pdet Qmax in this study, we found that all male and female patients had Pdet max or Pdet Qmax greater than 10 cmH_2_O, except for one male and two female patients. We think that the contribution of sustained intra-abdominal pressure may be small in our patients. It is usually impossible for patients with advanced MSA to urinate during pressure flow studies using primarily intra-abdominal pressure. Patients with advanced MSA usually have less intra-abdominal pressure than healthy people. We often experience cases of patients with advanced MSA who are unable to cough properly during urodynamic studies.

It should be noted that detrusor contractility is not equivalent to Pdet itself (17). Detrusor contractility should be evaluated by Pdet and urinary flow rate (11). In general, the contractility of a muscle is modeled by the Hill equation in the form of a hyperbolic relation between contraction velocity and force exerted by the muscle (18). In detrusor contraction, the force exerted by the detrusor muscle is equivalent to Pdet and detrusor shortening velocity which is estimated by urinary flow. Therefore, Watt’s Factor correlates with detrusor pressure and urinary flow. The detrusor contractility line of the Schäfer nomogram can be obtained by changing the size of WF, drawing a graph of multiple constant WF values, and approximating the obtained downward-sloping curve with a straight line. It has been shown that WF and BCI are significantly positively correlated (17). The currently available index estimating detrusor contractility depends on Pdet and urinary flow and Pdet alone is not an accurate measure of detrusor contraction.

It should discuss the utility of present findings in clinical practice of MSA. Although the evaluation of detrusor contractility is essential in the diagnosis of MSA, very few studies evaluated detrusor contractility. It is impossible to differentiate detrusor underactivity and bladder outlet obstruction by measuring the PVR alone. The established evaluation of detrusor contractility might be important to estimate the disease progression because PVR increases as the disease progresses (19). The relationships between detrusor contractility and other autonomic functions and motor functions might also be important to elucidate the clinical characteristics of MSA.

There are many limitations to this study. It is true that we excluded patients with MSA who showed obstructed patterns in the UDS to better clarify the relationships between bladder contractility and PVR/VOID%. However, it is not uncommon that MSA males may have comorbid urological complications such as benign prostatic hyperplasia which usually results in bladder outlet obstruction. Therefore, excluding obstructed patients might have biased the correlation. Additionally, because both bladder contractility and BOOI are evaluated by Pdet Qmax and Qmax, it might be difficult to separately evaluate BOOI and bladder contractility (8). Furthermore, the formula of BOOI is not validated in females.

In addition, it is usually difficult to perform a UDS in patients with MSA who have a severely deteriorated activity of daily living (ADL).

Furthermore, this study included patients with MSA who could void during the pressure-flow study. Because the insertion of the catheter into the urethra significantly increases urethral resistance, patients with MSA and severely impaired bladder contractility cannot void during the pressure-flow study. Therefore, our study excluded patients with MSA and severely impaired bladder contractility.

With respect to the method of UDS, because UDS is performed in a seated position, it might be non-physiological for a group of male patients who used to void in a standing position. The infusion rate of 50 mL/min may be a little fast for neurogenic patients, which might affect the present result.

Nevertheless, this study might be able to provide an appropriate formula representing bladder contractility in male patients with MSA which might help evaluate voiding dysfunction in multiple system atrophy.

## Conclusion

Although currently used formulas such as Pdet Qmax + 5Qmax might be appropriate for male MSA patients, formulas for female patients need further evaluation.

## Data Availability

The raw data supporting the conclusions of this article will be made available by the authors, without undue reservation.

## Author’s note

A summary of this research is posted on the website (https://www.m.chibau.ac.jp/dept/neurol/research/clinical/) in an opt-out format.
